# Identification of Z-Tyr-Ala-CHN_2_, a Cathepsin L Inhibitor with Broad-Spectrum Cell-Specific Activity against Coronaviruses, including SARS-CoV-2

**DOI:** 10.3390/microorganisms11030717

**Published:** 2023-03-10

**Authors:** Jordi Doijen, Koen Temmerman, Christel Van den Eynde, Annick Diels, Nick Van den Broeck, Michiel Van Gool, Inha Heo, Steffen Jaensch, Marleen Zwaagstra, Mayra Diosa Toro, Winston Chiu, Steven De Jonghe, Pieter Leyssen, Denisa Bojkova, Sandra Ciesek, Jindrich Cinatl, Lore Verschueren, Christophe Buyck, Frank Van Kuppeveld, Johan Neyts, Marnix Van Loock, Ellen Van Damme

**Affiliations:** 1 Janssen Pharmaceutica NV, Turnhoutseweg 30, 2340 Beerse, Belgium; 2 Charles River Laboratories, Turnhoutseweg 30, 2340 Beerse, Belgium; 3 Janssen Cilag S.A., C/Jarama 75A, 45007 Toledo, Spain; 4 Faculty of Veterinary Medicine, Yalelaan 1, Virology Division, Department of Biomolecular Health Sciences, Infectious Diseases and Immunology, Utrecht University, 3584 Utrecht, The Netherlands; 5 Laboratory of Virology and Chemotherapy, Herestraat 49, Rega Institute for Medical Research, Department of Microbiology, Immunology and Transplantation, KU Leuven, 3000 Leuven, Belgium; 6 Institute for Medical Virology, University Hospital, Paul-Ehrlich-Str. 40, Frankfurt University, 60596 Frankfurt am Main, Germany

**Keywords:** cathepsin L inhibitor, coronavirus, in vitro, phenotypic screening, SARS-CoV-2

## Abstract

The ongoing COVID-19 pandemic, caused by severe acute respiratory syndrome coronavirus 2 (SARS-CoV-2), is partly under control by vaccination. However, highly potent and safe antiviral drugs for SARS-CoV-2 are still needed to avoid development of severe COVID-19. We report the discovery of a small molecule, Z-Tyr-Ala-CHN_2_, which was identified in a cell-based antiviral screen. The molecule exerts sub-micromolar antiviral activity against SARS-CoV-2, SARS-CoV-1, and human coronavirus 229E. Time-of-addition studies reveal that Z-Tyr-Ala-CHN_2_ acts at the early phase of the infection cycle, which is in line with the observation that the molecule inhibits cathepsin L. This results in antiviral activity against SARS-CoV-2 in VeroE6, A549-hACE2, and HeLa-hACE2 cells, but not in Caco-2 cells or primary human nasal epithelial cells since the latter two cell types also permit entry via transmembrane protease serine subtype 2 (TMPRSS2). Given their cell-specific activity, cathepsin L inhibitors still need to prove their value in the clinic; nevertheless, the activity profile of Z-Tyr-Ala-CHN_2_ makes it an interesting tool compound for studying the biology of coronavirus entry and replication.

## 1. Introduction

Most individuals infected with severe acute respiratory syndrome coronavirus 2 (SARS-CoV-2) are protected from progression to severe disease and death by vaccination. However, some patient populations with underlying conditions do not qualify for [1] or do not have access to the vaccine [2]. Moreover, protection by vaccination is relatively short-lived due to waning antibody levels at 6–9 months post vaccination [3], and because of reduced neutralization of variants by vaccination-elicited antibodies [4,5,6,7,8,9,10]. In addition, the current vaccines against SARS-CoV-2 will most likely not protect against future coronavirus outbreaks. Therefore, there is a need for safe therapeutic agents. To this end, several drugs have been investigated, including remdesivir [11], molnupiravir [12,13], favipiravir [14] (nucleoside analogues), tocilizumab and sarilumab (interleukin-6 receptor modulators) [15,16], and ritonavir-boosted nirmatrelvir (main protease inhibitor (M^pro^)) [17], some of which are also being investigated as combination therapies [18,19]. 

SARS-CoV-2 makes use of human angiotensin-converting enzyme II (hACE2) expressed in the airway to infect its host [20]. The viral spike (S) protein recognizes and binds to hACE2, whereafter the spike protein is primed either at the cell surface by transmembrane protease serine subtype 2 (TMPRSS2) or within the endosomal pathway by cathepsin L [21,22,23]. This priming event facilitates cell fusion, which in turn results in release of the viral content into the host cell [24]. The same two proteases are involved in S protein priming and entry of SARS-CoV-1 [23]. This makes hACE2, TMPRSS2, and cathepsin L key targets for blocking SARS-CoV-1 and SARS-CoV-2 entry into cells [23]. As such, efforts have been made to find drugs that target these proteins [25,26,27,28,29,30,31], such as the small molecules K777 and camostat mesylate, which respectively target cathepsin L [29] and TMPRSS2 [32]. 

One approach to discover new coronavirus antivirals is phenotypic screening, which is a cell-based, untargeted, and unbiased screening approach to discover primary hit compounds that lend themselves to further optimization [33]. Phenotypic screening can identify compounds that act on different stages of the virus replication cycle (entry-stage blockers, host-targeting antivirals, protease inhibitors, and replication blockers) [34]. Typically, after hit identification in phenotypic screening runs, the target of the hit must be identified by more specialized assays. 

Here, we report the discovery of Z-Tyr-Ala-CHN_2_ from a phenotypic screen. The molecule acts early in the viral replication cycle by targeting cathepsin L. Due to its cell-specific activity, the compound is a valuable research tool for studying the entry and replication mechanisms of SARS-CoV-2 and other coronaviruses. 

## 2. Materials and Methods

### 2.1. Cell Lines and Viruses 

VeroE6-eGFP cells were generated and maintained as described previously [35]. A549-hACE2 cells were purchased from InvivoGen (San Diego, CA, USA) and were cultivated in RPMI-1640 medium (Sigma, St Louis, MO, USA) supplemented with 10% (*v*/*v*) heat-inactivated fetal calf serum (HIFCS; Biowest, Nuaillé, France) and 0.5 µg/mL puromycin (Gibco, Waltham, MA, USA). HeLa-hACE2 cells were purchased from Creative Biogene (New York, NY, USA). The cells were maintained in Dulbecco’s Modified Eagle Medium (DMEM; Lonza, Basel, Switzerland) supplemented with 10% (*v*/*v*) HIFCS and 0.5 µg/mL puromycin. Both A549-hACE2 and HeLa-hACE2 antiviral assays were performed using medium with the same composition as the respective culture media but without puromycin. Caco-2 cells were maintained by Frankfurt University in Minimal Essential Medium (MEM; Sigma, St Louis, MO, USA) supplemented with 10% (*v*/*v*) FCS, 100 IU/mL of penicillin, and 100 µg/mL of streptomycin (all from Sigma, St Louis, MO, USA) following established procedures [36]. VeroE6 cells (ATCC CRL-1586) were maintained by Utrecht University in DMEM (Gibco, Waltham, MA, USA) supplemented with 10% (*v*/*v*) FCS, 20 mM of HEPES (Lonza, Basel, Switzerland), 0.075% sodium bicarbonate (Gibco, Waltham, MA, USA), penicillin (100 IU/mL), and streptomycin (100 IU/mL) following established protocols [37,38]. VeroE6 TMPRSS2 cells were obtained from the laboratory of Bart Haagmans (Rotterdam, the Netherlands) and were cultured by Utrecht University using the same medium composition as VeroE6, but supplemented with hygromycin.

Virus stocks were generated and titrated in the VeroE6 cell line (SARS-CoV-2 Belgium (strain BetaCov/Belgium/GHB-03021/2020 B.1 lineage A, SARS-CoV-2 Omicron variant (strain B.1.1.529-BA.1), SARS-CoV-1 (strain human coronavirus 19/Germany/FrankfurtFFM1/2020)) or in the Huh7 cell line (human coronavirus 229E) (HCoV-229E (strain AlphaCoV/ATCC/VR-740))). Titrations were performed using an endpoint dilution assay by reverse transcriptase quantitative polymerase chain reaction (RT-qPCR), microscopic cytopathic effect (CPE) scoring, or immunofluorescent staining. Titers were calculated using the Reed–Muench method and reported in tissue culture infectious doses per mL of sample (TCID_50_/mL).

### 2.2. Antiviral Compounds 

The half-maximal effective concentration (EC_50_) or half-maximal cytotoxic concentration (CC_50_) of Z-Tyr-Ala-CHN_2_ was compared across cell lines and coronaviruses with other antiviral agents. These compounds were PF-0083523 (intravenous M^pro^ inhibitor; Pfizer, New York, NY, USA), nirmatrelvir/PF-07321332 (oral M^pro^ inhibitor; Pfizer, New York, NY, USA), apilimod (Merck, Darmstadt, Germany), K777 (BOC Sciences, New York, NY, USA), remdesivir (Merck, Darmstadt, Germany), brefeldin A (Santa Cruz Biotechnology, Dallas, TX, USA), and molnupiravir (Bio-Connect BV, Huissen, The Netherlands). Compounds were dissolved in 100% dimethyl sulfoxide (DMSO), stored at room temperature, and eventually diluted to a defined dosage in experimental medium. The final in-assay DMSO concentration did not exceed 0.4% (*v*/*v*).

### 2.3. Antibodies, Cellular Counterstains, and Staining Buffers for Immunofluorescence and LysoTracker Assays

Viral S protein was detected by interaction with anti-spike S1 monoclonal antibody (anti-rabbit), purchased from Sino Biological (Houston, TX, USA), used at 1:1000 or 1:2000 dilution. Primary anti-double-stranded RNA (dsRNA) monoclonal antibody J2 (anti-mouse) was purchased from SCICONS (Jena Bioscience, Jena, Germany) and diluted to 1:2000 or 1:2500 in staining buffer. Polyclonal immunoglobulin G (IgG) secondary antibodies, conjugated to Alexa Fluor 488 (goat anti-mouse) or Alexa Fluor 568 (goat anti-rabbit) were purchased from Invitrogen (Waltham, MA, USA) and were used at dilutions of 1:500 or 1:800. Hoechst 33,342 for nucleus identification and HCS CellMask™ Deep Red Stain for visualization of the entire cell structure were purchased from Invitrogen (Waltham, MA, USA). These stains were used at a dilution of 1:1000 or 1:2800 and 1:10,000, respectively. Buffers used for permeabilization, blocking, and staining were prepared in a sterile solution of 1% bovine serum albumin (BSA, Sigma, St Louis, MO, USA) in Dulbecco’s phosphate buffered saline (DPBS; Invitrogen, Waltham, MA, USA). Goat serum (Sigma, St Louis, MO, USA) at a concentration of 5 % (*v*/*v*) was used for blocking, and Triton X-100 (Sigma, St Louis, MO, USA) was used at 0.1 % (*v*/*v*) as a permeabilizing agent.

### 2.4. Phenotypic Screening and In Vitro Assays

#### 2.4.1. SARS-CoV-2 Antiviral Assay in VeroE6-eGFP Cells 

The screen was conducted at KU Leuven as published [39]. In brief, VeroE6-eGFP cells were seeded on 384-well assay plates that were pre-spotted with compound. After 24 h of incubation at 37 °C, cells were infected with SARS-CoV-2 at a multiplicity of infection (MOI) of 0.001. The plates were then incubated for an additional 96 h at 37 °C. The plates were then imaged in an ArrayScan™ XTI imager (Thermo Fisher Scientific, Waltham, MA, USA) to determine the percentage of infected cells based on the spot total area parameter. Genedata Screener^®^ v.17 software (Basel, Switzerland) was used to analyze the compound activity data. Dose-response curves were plotted and EC_50_ values were calculated using GraphPad Prism v8 (San Diego, CA, USA). 

#### 2.4.2. SARS-CoV-2 Viral RNA Copy Determination in A549-hACE2 Cells 

Eight thousand cells per well were seeded on pre-spotted compound plates 24 h before inoculation with SARS-CoV-2 at MOI 0.1. After incubation for 2 h at 37 °C, cell supernatants were removed, and the plates were washed once with DPBS. Plates were re-filled with compounds diluted in culture medium to the same concentration as before the wash. Plates were incubated for an additional 48 h in a humidified incubator at 37 °C and 5% CO_2_. Supernatants were collected and treated with MagNA Pure 96 external lysis buffer (Roche Diagnostics, Basel, Switzerland) and used as input for automated RNA extraction using the MagNA Pure 96 instrument and the complementary DNA and Viral NA Small Volume Kit (Roche Diagnostics, Basel, Switzerland). Extracted RNA was used as a template in one-step RT-qPCR in a master mix containing LightCycler^®^ Multiplex RNA Virus Master (Roche Life Science, Penzberg, Germany) components and a SARS-CoV-2 primer/probe set, ordered from Integrated DNA Technologies (Leuven, Belgium), targeting the viral nucleocapsid genetic sequence. Primer and probe sequences were as follows: Forward: 5′-GACCCCAAAATCAGCGAAAT-3′, Reverse: 5′-TCTGGTTACTGCCAGTTGAATCTG-3′, Probe: 5′-FAM-ACCCCGCATTACGTTTGGTGGACC-BHQ1–3′. In-house prepared SARS-CoV-2 nucleocapsid RNA was included as a standard to allow virus copy quantitation. RT-qPCR itself was performed using the LightCycler 480 (Roche Life Science, Penzberg, Germany) and consisted of the following cycling steps: reverse transcription; 50 °C—10 min, denaturation, and polymerase activation; 95 °C—30 s, followed by 45 cycles of denaturation; 95 °C—5 s and annealing plus extension; 60 °C—30 s. Resulting copy numbers, as well as the viral yield reduction (log_10_ scale), were plotted in function of the compound concentration using GraphPad Prism v8.

#### 2.4.3. Immunofluorescence-Based Antiviral Assays 

A549-hACE2 and HeLa-hACE2 cells were infected with SARS-CoV-2 at an MOI of 0.1 in 1536-well Aurora plates (Aurora Microplates, Whitefish, MT, USA) at 3000 cells/well and 384-well collagen-coated plates (Cell Carrier Ultra, Perkin Elmer, Waltham, MA, USA) at 4000 cells/well, respectively. For the A549-hACE2 cells, plates were pre-spotted with compound before adding the cells and virus, whereas for the HeLa-hACE2, compound was added to 24-h pre-seeded cells prior to adding the inoculum. For SARS-CoV-1 antiviral testing, A549-hACE2 cells (10,000 cells/well) were seeded and inoculated simultaneously in pre-spotted 384-well compound plates (Cell Carrier Ultra) at MOI 0.1. Antiviral activity against HCoV-229E in HeLa-hACE2 cells was evaluated in pre-spotted 384-well assay plates (Cell Carrier Ultra). Eight thousand cells per well and virus were also added at the same time, but the MOI was 0.8 in this assay design. Plates were kept in a humidified incubator for 24 h at 37 °C (SARS-CoV-2 and SARS-CoV-1) or at 35 °C (HCoV-229E). After incubation, infected cells were fixed with methanol-free formaldehyde (Polysciences, Warrington, PA, USA) at a final concentration of 3–4% (*v*/*v*) for 20 min. Subsequently, the assay plates were treated with permeabilizing and staining solutions, to ultimately image the viral S protein, and/or dsRNA viral marker with nucleus and cell outline stains by high-throughput confocal microscopy (Cell Voyager 8000, Yokogawa, Musashino, Tokyo, Japan). The generated high-content imaging (HCI) data were exported to the Phaedra HCI analysis software [40] for further analysis in order to calculate the percentage of infected cells.

#### 2.4.4. Immunofluorescence-Based Antiviral Assay Using LysoTracker Dye to Detect Accumulation of Acidic Organelles 

Two thousand HeLa-hACE2 cells per well were seeded and infected with SARS-CoV-2 at MOI 0.1 in 1536-well plates (Aurora Microplates) pre-spotted with compound. After incubation at 37 °C for 24 h, LysoTracker reagent (LysoTracker DND-99, Invitrogen, Waltham, MA, USA) was added to the plates at a final concentration of 5 µM. The plates were then fixed and subjected to subsequent permeabilization, blocking, and staining for dsRNA, cell outline, and nucleus. Plates were imaged on the Cell Voyager 8000 Yokogawa confocal microscope. The LysoTracker signal and the percentage of dsRNA-positive cells were analyzed using Phaedra HCI analysis software. The LysoTracker signal was plotted relative to the blank signal and was defined as positive for acidic organelle accumulation if a significant increase in signal was observed relative to the baseline (virus control). 

#### 2.4.5. Time-of-Addition (TOA) Assay 

For HeLa-hACE2, 4000 cells/well were seeded in 384-well plates and incubated for 24 h at 37 °C and 5% CO_2_. On day 2, the medium was washed away, whereafter the cells were infected with SARS-CoV-2 (MOI 4). After a 1 h incubation at 37 °C, the plate was washed five times with medium using a 405 TS Washer (Biotek, Winooski, VT, USA). After the final wash, the cells in medium were incubated for an additional 7 h at 37 °C and 5% CO_2_. At different time points (0, 2, and 4 h post infection (hpi)), compound was added to the infected wells at a final compound concentration of 2.5–5 µM using the VIAFLO 384 (Integra Biosciences, Zizers, Switzerland). To wells that received compound at 0 hpi, the compound was readministered after the wash. Wells that were not exposed to compound prior to or during infection received a similar concentration of DMSO during infection. At 8 hpi, the cells were fixated, stained for dsRNA, and imaged as described above. Data were analyzed using the Phaedra HCI software. The percentage of dsRNA-positive cells was used as a measure of viral inhibition. Data were visualized using GraphPad Prism v8.

#### 2.4.6. Pseudotyped Virus Neutralization Assay in VeroE6 Cells 

Pseudotype vesicular stomatitis virus (VSV) carrying SARS-CoV-2 S protein (SARS2pp) or VSV fusion glycoprotein G (VSV-Gpp) were produced as previously described [41,42].

VeroE6 cells or VeroE6 cells ectopically expressing TMPRSS2 were seeded at a cell density of 25,000 cells/well in 96-well plates and cultured overnight. Compounds and Regeneron monoclonal antibodies (positive control; Regeneron Pharmaceuticals, Tarrytown, NY, USA), were serially diluted in DMEM supplemented with 1% (*v*/*v*) FCS and inoculated on confluent VeroE6 monolayers. Compounds were incubated at 37 °C for 1 h prior to infection with VSV-Gpp or VSV pseudotype virus carrying the SARS2pp and expressing firefly luciferase. After infection, cells were further incubated for 24 h, and the relative luciferase units (RLU) were determined using the GloMax Explorer luminometer (Promega, Madison, WI, USA) and D-luciferin as a substrate. Data are presented as the percentage of RLU, calculated as the ratio between RLU in the presence of compound vs. the RLU of DMSO-treated control. The EC_50_ values were calculated using GraphPad Prism v8. 

#### 2.4.7. Antiviral Assessment in Caco-2 Cells by Visual CPE Scoring 

Virus-induced CPE was used to assess antiviral activity in Caco-2 cells at Frankfurt University. In brief, confluent layers of Caco-2 cells cultured for 72 h on 96-well plates (50,000 cells/well) were challenged with SARS-CoV-2 at an MOI of 0.01. Cells were incubated for 48 h, after which the CPE was visually scored independently by two laboratory technicians. All assays were performed six times independently in triplicate. Data were analyzed using GraphPad Prism v8 to calculate the EC_50_. 

#### 2.4.8. Chemiluminescence-Based and Colorimetric Cytotoxicity Assays

In parallel with the antiviral activity assessment, a cytotoxicity counter screen was performed in either VeroE6-eGFP, A549-hACE2, HeLa-hACE2, Caco-2, VeroE6, and VeroE6 TMPRSS2 cells. The cytotoxicity counter screen followed the same workflow as the corresponding antiviral assay, except that inoculum was substituted for cell culture medium. After an assay-specific incubation period at 37 °C, the endpoint cytotoxicity assay was performed. For VeroE6-eGFP, A549-hACE2, and HeLa-hACE2 cells, one-step ATPlite reagent (Perkin Elmer, Waltham, MA, USA) was prepared per the manufacturer’s instructions and added to the assay plates in a 1:1 volume ratio. Plates were incubated at room temperature in the dark on an orbital shaker at 700 rpm for 2 min. ATP-generated chemiluminescence was measured using the ViewLux™ plate reader (Perkin Elmer, Waltham, MA, USA). The resulting data were normalized in Genedata Screener v.18. Cytotoxicity in Caco-2 cells was assessed in the absence of virus using the Rotitest^®^ Vital (Roth, Karlsruhe, Germany) according to the manufacturer’s instructions. Cytotoxicity in VeroE6 and VeroE6 TMPRSS2 cells was measured using the CellTiter 96^®^ AQ_ueous_ One Solution Cell Proliferation MTS colorimetric assay (Promega, Madison, WI, USA) according to the manufacturer’s instructions. For each assay, dose-response curves were created using GraphPad Prism v8, and CC_50_ values were calculated.

#### 2.4.9. Cathepsin L Inhibition Assay 

Cathepsin L inhibition was measured using an enzymatic cathepsin L assay performed at Eurofins. Z-Tyr-Ala-CHN_2_, K777, reference compound (leupeptin), and control (water) were pre-incubated for 5 min at room temperature with 0.02 mU cathepsin L in a buffer containing 45 mM sodium acetate, 0.9 mM ethylenediaminetetraacetic acid, 4.5 mM dithiothreitol, and 0.0045% Brij^®^35 (pH 5.5); 15 µM of the substrate (Z-Phe-Arg AMC) was added, and the cells were incubated for 30 min at room temperature. The fluorescence intensity was measured at λex = 340 nm and λem = 460 nm using a microplate reader (Envision, Perkin Elmer, Waltham, MA, USA). Measurements were taken at t = 0 and t = 30 min after addition of the substrate. Enzyme activity was determined by subtracting the signal measured at t = 0 from the signal at t = 30. 

#### 2.4.10. Air–Liquid Interface (ALI) Cultures 

Pooled donor human nasal epithelial cells (HNECs) grown in ALI format were obtained from Epithelix (Geneva, Switzerland) as a fully differentiated culture and maintained in-house for a week before experimentation. At the start of the experiment, compounds and vehicle (DMSO 0.2%) in MucilAir™ medium from Epithelix were added to the basal compartment of the ALI inserts in 24-well plates and incubated for 1 h. Brefeldin 0.3 µM was used as a toxicity control and PF-07321332 at 2.5 µM was used as an antiviral control. Next, inserts were infected with SARS-CoV-2 (MOI 0.1) for 1 h at 37 °C. The inoculum was washed away using DPBS and cultures were incubated at 37 °C for 72 h. At the 48 h time point, compounds were refreshed. At 24, 48, and 72 hpi, the inserts were apically washed by adding DPBS to the apical side and incubating for 15 min at 37 °C. The apical washes were collected and stored at −80 °C. Automated RNA extraction was performed on these apical washes using the MagNA Pure 96 instrument followed by one-step RT-qPCR. The same protocols, kits, primers, and probe set were used as in the section SARS-CoV-2 Viral RNA Copy Determination in A549-hACE2 Cells. Toxicity was assessed by exposing non-infected inserts to the same concentration of compound as in the antiviral setting over the same time course (72 h). For this purpose, a voltmeter (EVOM3 from World Precision Instruments, Sarasota, FL, USA) that measures transepithelial electrical resistance, which is representative for the cell layer’s integrity or health, was used. To perform the measurement, DPBS was added to the apical compartment; this was carefully removed once the measurement was taken. Both activity and toxicity data were further analyzed using GraphPad Prism v8.

#### 2.4.11. Dose-Response Curve Fitting and EC_50_ and CC_50_ Calculations 

High-content imaging data were analyzed using in-house developed pipelines in the Phaedra HCI analysis software. The percentage of infected cells, either defined by Spike or dsRNA positivity, was calculated, and the values were normalized to % effect. For each assay, technical replicates were averaged within each experiment, and the mean and standard deviation (SD) across independent experiments are presented. Dose-response curves were generated in GraphPad Prism v8. When experimental controls were used to normalize the data, the (Inhibitor) vs. normalized response–variable slope was chosen as the model for non-linear fitting. For viral RNA copy determination assays, the top of the curve was constrained to 0 while the bottom was not constrained (3 parameter fit). From these fits, EC_50_ and CC_50_ values were calculated using the same software.

## 3. Results

### 3.1. Z-Tyr-Ala-CHN_2_ Shows Antiviral Activity against SARS-CoV-2 in VeroE6-eGFP Cells

**Figure 1:**
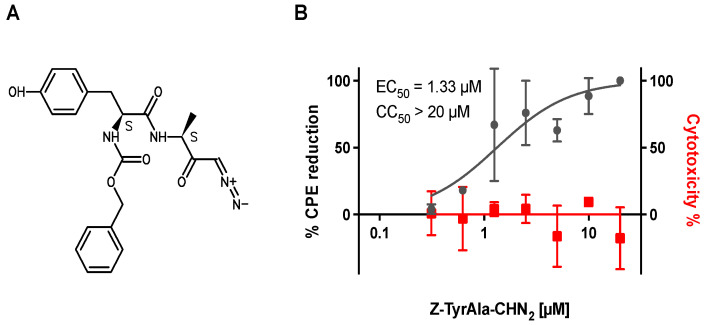
(**A**) Structure of Z-Tyr-Ala-CHN_2_. (**B**) Antiviral activity of Z-Tyr-Ala-CHN_2_ against SARS-CoV-2 from the primary screen performed in VeroE6-eGFP. The SpotTotalAreaCh2 of the green fluorescent protein (GFP) signal was used to quantify CPE reduction. Toxicity was determined on the same cell line and was quantified using ATPlite. The results represent data from two to three independent experiments. CPE, cytopathic effect; CC_50_, half-maximal cytotoxic concentration; EC_50_, half-maximal effective concentration; eGFP; enhanced GFP; GFP, green fluorescent protein; SARS-CoV-2, severe acute respiratory syndrome coronavirus 2.

Z-Tyr-Ala-CHN_2_ was identified as a primary hit in a phenotypic screen against SARS-CoV-2 (B.1) in VeroE6-eGFP cells with an EC_50_ of 1.33 µM against SARS-CoV-2 and a CC_50_ > 20 µM (selectivity index (SI) > 15) (Figure 1). The viral polymerase blocker remdesivir was included as a reference compound, as at the time of the screen, it was the most prominent SARS-CoV-2 antiviral [43] and had an EC_50_ of 1.34 µM and CC_50_ > 100 µM (not shown). A set of available analogues of Z-Tyr-Ala-CHN_2_ was assessed as part of a hit expansion exercise, but none of the analogues had antiviral activity against SARS-CoV-2 (EC_50_ > 20 µM) (Figure S1). 

### 3.2. Confirmation of Z-Tyr-Ala-CHN_2_ Activity in a Viral RNA Yield Assay 

**Figure 2:**
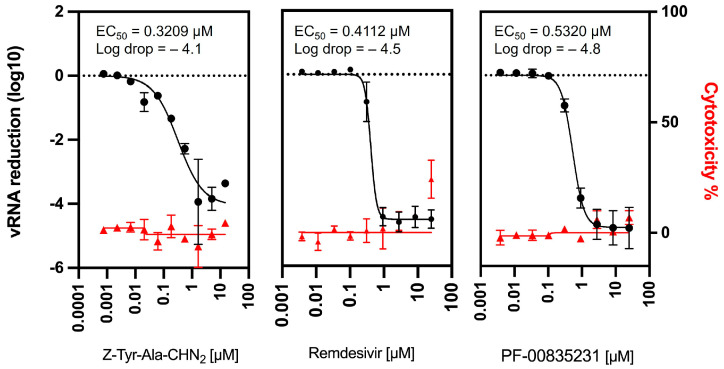
Comparison of viral RNA reduction in the supernatant of SARS-CoV-2-infected A549-hACE2 cells using Z-Tyr-Ala-CHN_2_ (*n* = 2), remdesivir (*n* = 3), and PF-00835231 (*n* = 3). Toxicity was determined using ATPlite on uninfected A549-hACE2 cells and is based on two independent experiments. No significant differences (*p* < 0.05) in compound potency and log drop were observed using Welch’s unpaired t testing (assuming normality of data).

To confirm the antiviral activity of Z-Tyr-Ala-CHN_2_ against SARS-CoV-2, we tested its effect on viral RNA yield. To this end, RT-qPCR was performed at 48 hpi on supernatant of A549-hACE2 cells infected with SARS-CoV-2. Remdesivir and the M^pro^ inhibitor PF-00835231 were included as reference compounds since both have a well-described mechanism of action and are known to reduce SARS-CoV-2 RNA levels [43,44]. Z-Tyr-Ala-CHN_2_ did not show cytotoxicity and reduced SARS-CoV-2 RNA (~4-log reduction) in the supernatant, similarly to remdesivir (~4.5-log reduction) and PF-00835231 (~4.8-log reduction) (Figure 2). The potency of the three compounds was also comparable, with an EC_50_ of 0.32, 0.41, and 0.53 µM for Z-Tyr-Ala-CHN_2_, remdesivir, and PF-00835231, respectively. 

### 3.3. Assessment of Broad-Spectrum Antiviral Activity of Z-Tyr-Ala-CHN_2_ against SARS-CoV-1 and HCoV-229E

**Figure 3:**
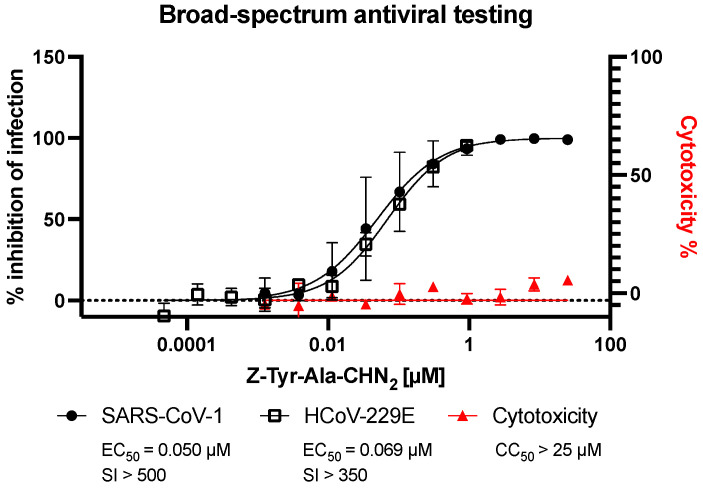
Antiviral activity of Z-Tyr-Ala-CHN_2_ against SARS-CoV-1 (black, full circles) in A549-hACE2 and against HCoV-229E (black, empty squares) in HeLa-hACE2. Infection of SARS-CoV-1 was detected using an anti-spike antibody, whereas infection against HCoV-229E was detected using an anti-dsRNA antibody. The mean ± SD values are shown from two to three independent experiments. Cytotoxicity determined in uninfected A549-hACE2 using ATPlite (red, full triangles) represents the mean ± SD of two independent experiments. HCoV-229E, human coronavirus 229E; SD, standard deviation; SI, selectivity index.

Next, the antiviral activity of Z-Tyr-Ala-CHN_2_ was assessed against the closely related beta coronavirus SARS-CoV-1 and the alpha coronavirus HCoV-229E. In these assays, viral infection was monitored using intracellular staining of spike or dsRNA. Z-Tyr-Ala-CHN_2_ showed activity against SARS-CoV-1 and HCoV-229E (Figure 3), with EC_50_ of 0.050 and 0.069 µM, respectively, which suggests the compound is a broad-spectrum coronavirus antiviral.

### 3.4. Antiviral Activity of Z-Tyr-Ala-CHN_2_ across Different Cellular Backgrounds 

**Figure 4:**
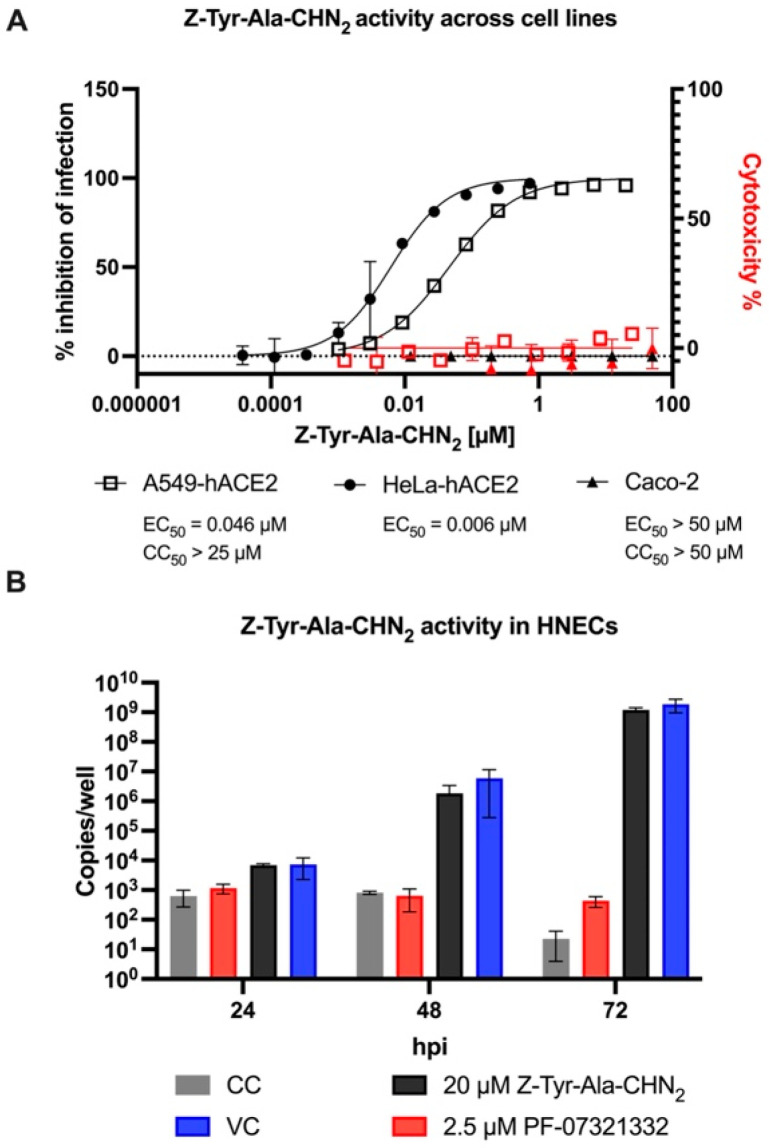
(**A**) The antiviral activity of Z-Tyr-Ala-CHN_2_ against SARS-CoV-2 in A549-hACE2 (black, empty squares), HeLa-hACE2 (black, full circles), and Caco-2 (black, full triangles) cells. The mean values ± SD are shown of three (A549-hACE2 and HeLa-hACE2) to six (Caco-2) independent experiments. Cytotoxicity was determined in A549-hACE2 (red, empty squares) using ATPlite and in Caco-2 (red, full triangles) using Rotitest^®^ Vital. The mean values ± SD are shown from two (A549-hACE2) to six (Caco-2) independent experiments. (**B**) Z-Tyr-Ala-CHN_2_ does not inhibit SARS-CoV-2 infection in an upper respiratory tract model. HNEC ALI cultures were infected with SARS-CoV-2 and treated with PF-07321332 at 2.5 µM (red) and Z-Tyr-Ala-CHN_2_ at 20 µM (black). Cell control (CC; (gray bars, DMSO 0.2%); Virus control (VC; blue bars, MOI 0.1 without compound). The mean ± SD from within one representative experiment is shown. Within each experiment, conditions are performed in duplicate. ALI, air-liquid interface; HNEC, human nasal epithelial cell; MOI, multiplicity of infection.

To rule out cell-specific effects of Z-Tyr-Ala-CHN_2_ against SARS-CoV-2, its antiviral activity was tested in A549-hACE2 and HeLa-hACE2 cells using intracellular staining of S protein or dsRNA, and in Caco-2 cells using visual CPE scoring. Z-Tyr-Ala-CHN_2_ was found to potently inhibit infection in both A549-hACE2 and HeLa-hACE2 cells. However, no effect was observed in Caco-2 cells (Figure 4A; A549-hACE2: EC_50_, 0.046 µM; CC_50_, >25 µM; SI, >500; HeLa-hACE2: EC_50_, 0.006 µM; Caco-2: EC_50_, >50 µM; CC_50_, >50 µM). 

To further investigate the cell dependency of the observed effect, we also assessed the activity of Z-Tyr-Ala-CHN_2_ in a translational model of the upper respiratory tract. This model was established by growing HNECs in ALI [45] and by infecting the apical compartment of the culture with an early Belgian isolate (Lineage A, B.1) of SARS-CoV-2. Infection was monitored by measuring viral RNA copies in the apical culture supernatants for three days (Figure 4B). The M^pro^ inhibitor nirmatrelvir/PF-07321332 was included as reference antiviral as ritonavir-boosted nirmatrelvir is currently the standard of care for mild to moderate COVID-19 infection [46]. Since it can be administered orally, it is a more relevant reference than remdesivir in this system. Z-Tyr-Ala-CHN_2_ did not show antiviral activity at 20 µM whereas PF-07321332 at 2.5 µM did. Similarly, when the experiment was performed using the SARS-CoV-2 Omicron variant (B.1.1.529-BA.1), no inhibitory activity of Z-Tyr-Ala-CHN_2_ was observed (Figure S2).

Table 1 summarizes the activity of Z-Tyr-Ala-CH_2_ across different coronaviruses and cellular backgrounds, as well as how it compared to remdesivir, K777, PF-00835231, and molnupiravir. K777 was included as it acts on an early step in the virus life cycle, namely, cathepsin L inhibition [29]. All the other drugs were included as they, or variants thereof, are on the market for treatment of COVID-19 infection.

K777 showed the lowest EC_50_ in HeLa-hACE2 and A549-hACE2 cells for all human coronaviruses tested (SARS-CoV-2, SARS-CoV-1, and HCoV-229E), followed by Z-Tyr-Ala-CHN_2_ with over ten times lower potency than K777. Thus, in these cellular backgrounds, Z-Tyr-Ala-CHN_2_ outperformed remdesivir, PF-00835231, and molnupiravir.

### 3.5. Assessment of the Mechanism of Action of Z-Tyr-Ala-CHN_2_

#### 3.5.1. TOA Assay 

**Figure 5:**
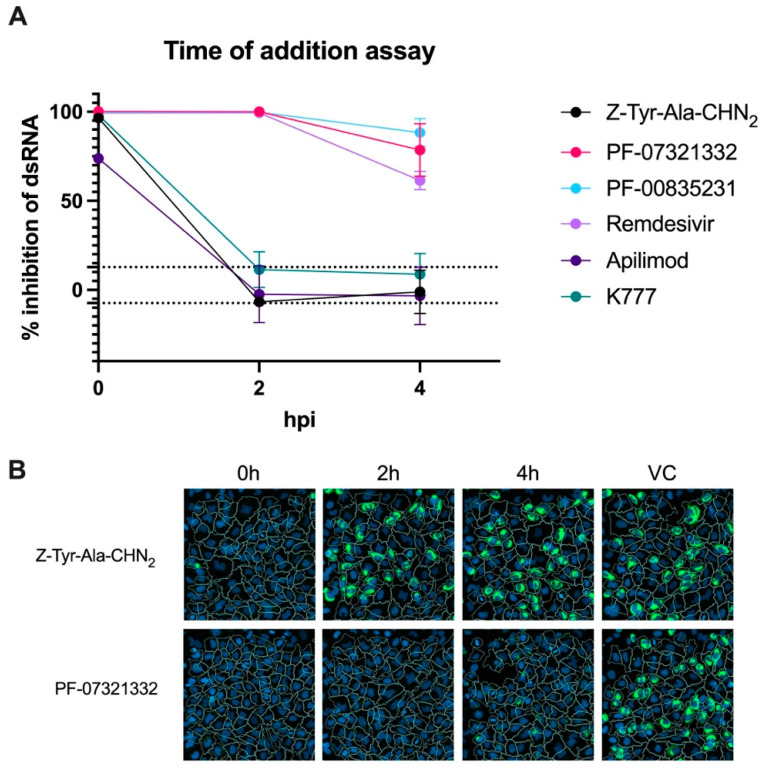
(**A**) TOA assay comparing the percentage inhibition of dsRNA formation in SARS-CoV-2-infected cells at 8 hpi by Z-Tyr-Ala-CHN_2_, remdesivir, PF-07321332 (nirmatrelvir), PF-00835231, K777, and apilimod added at 0, 2, and 4 hpi. In one of the runs, 2.5 µM of compound was used, whereas the other two runs used 5 µM of compound; both concentrations are known to result in ~100% inhibition of dsRNA in HeLa-hACE2 cells when added at 0 hpi. The mean values ± SD are shown from two independent experiments for Z-Tyr-Ala-CHN_2_, remdesivir, apilimod, and PF-00835231, and from three independent experiments for PF-07321332 and K777. The dotted horizontal lines represent the mean ±3 × SD of the virus control, indicative for the variation within the assay. (**B**) Microscopic images (20× objective) of representative wells treated with Z-Tyr-Ala-CHN_2_ or PF-07321332 at the indicated time points post infection. The Phaedra HCI analysis software was used to calculate cell outlines (white lines) using the CellMask Deep Red staining (channel not shown) and the nuclear staining (Hoechst 33342; blue). The dsRNA signal is shown in green. In the untreated/virus control condition, an average of 30% of the cells were dsRNA positive at 8 hpi. hpi, hours post infection; TOA, time of addition.

The lack of Z-Tyr-Ala-CHN_2_ activity in HNECs and Caco-2 cells demonstrates that there is a cell-specific antiviral effect and that it potentiates the involvement of a host target. To assess the mechanism of action of Z-Tyr-Ala-CHN_2_, we determined the point at which Z-Tyr-Ala-CHN_2_ affects the replication cycle of SARS-CoV-2 using an HCI-based TOA assay with intracellular dsRNA staining. The readout at 8 hpi was near the end of the first replication cycle but still before virion release [47,48]. The TOA assay showed that Z-Tyr-Ala-CHN_2_ antiviral activity was lost when administered at 2 h post virus infection or later, suggesting that the compound targets one of the early steps in the SARS-CoV-2 replication cycle (Figure 5A). A similar trend was seen for apilimod and K777, which were included as they block early steps in the virus replication cycle. In contrast, the inhibition of dsRNA formation by remdesivir, PF-07321332, and PF-00835231 was sustained until ~4 hpi, confirming that these compounds exert an effect at a later point in time (a viral polymerase blocker and two M^pro^ inhibitors, respectively) [43,44]. Figure 5B shows how the infection, measured using dsRNA staining, differs between Z-Tyr-Ala-CHN_2_ and PF-07321332 across time points at which the compound was administered. 

#### 3.5.2. Pseudotyped Virus Neutralization Assay in VeroE6 Cells

**Figure 6:**
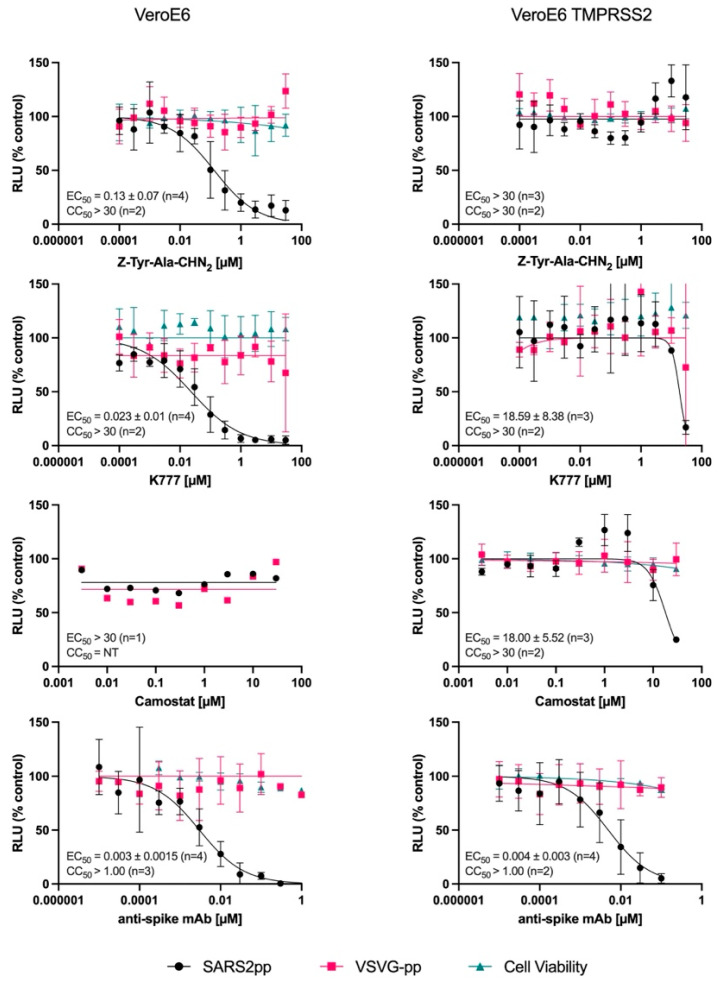
Pseudotyped SARS-CoV-2 neutralization assay in VeroE6 cells (left panel) and in VeroE6 TMPRSS2 cells (right panel). The Regeneron monoclonal antibody was included as a positive control because it can block entry in both cell types. VSV-Gpp was included to determine the specificity of the compounds. For infection with SARS2pp and VSV-Gpp, the mean ± SD values are shown for three to four independent experiments, whereas for cell viability, the mean ± SD values are shown for two to three independent experiments. Mean EC_50_ and CC_50_ values of the different compounds in the pseudotyped SARS-CoV-2 neutralization assay are given as well. NT, not tested; TMPRSS2, transmembrane protease serine subtype 2; VSV-Gpp, vesicular stomatitis virus fusion glycoprotein G; SARS2pp, SARS-CoV-2 S protein.

In the TOA assay, we observed that Z-Tyr-Ala-CHN_2_ is active early in the viral replication cycle. A pseudotyped virus neutralization assay was used to confirm an entry mode of action of Z-Tyr-Ala-CHN_2_ and to assess whether Z-Tyr-Ala-CHN_2_ acts on the endosomal entry route or on the TMPRSS2-dependent entry route. A Regeneron anti-spike monoclonal antibody was included as positive control as it can block the interaction between spike and hACE2, and thus can block entry independent of the entry route chosen by the pseudotyped virus. Like the Regeneron anti-spike monoclonal antibody, both Z-Tyr-Ala-CHN_2_ and K777 could neutralize SARS-CoV-2 pseudovirus (SARS2pp) entry in VeroE6 with EC_50_ values of 0.13 and 0.023 µM, respectively (Figure 6, left panel). Camostat mesylate, a TMPRSS2 blocker, was included to assess which entry route was used by the pseudotyped virus to enter the cell. The compound was not active in the assay in VeroE6 (EC_50_ > 30 µM) but was active (EC_50_ ~18 µM) when the assay was repeated in VeroE6 TMPRSS2 cells in which SARS2pp can enter via the TMPRSS2-dependent entry route (Figure 6, right panel). In contrast, the EC_50_ of K777 shifted from 0.023 µM to 18.6 µM when repeated on VeroE6 TMPRSS2 cells, while Z-Tyr-Ala-CHN_2_ was not active at all upon expression of TMPRSS2 in VeroE6. This observation suggests that Z-Tyr-Ala-CHN_2_ acts on the endosomal entry pathway. None of the compounds induced toxicity at the concentrations tested, as can be seen from the CC_50_ values in the figure.

Since the spike glycoprotein-carrying VSV is promiscuous and can infect many different cell types [49], VSV-Gpp was also included in the assay to assess whether the presumed entry blockers are specific for coronaviruses or instead have a more general mechanism of action. Since no activity was observed against VSV-Gpp for either one of the compounds **(Figure 6**; red curves), we can state that the compounds are specifically active against the entry of coronaviruses. 

#### 3.5.3. Z-Tyr-Ala-CHN_2_ Is a Cathepsin L Inhibitor That Does Not Cause Disruption of Lysosome Trafficking

Since Z-Tyr-Ala-CHN_2_ acts early in the SARS-CoV-2 replication cycle and activity was lost in the pseudotyped virus neutralization assay when TMPRSS2 was present, it is probable that the compound acts within the endolysosomal entry pathway. Moreover, the well-described cathepsin L inhibitor K777, had a similar activity profile in both the TOA assay and pseudovirus assay. Since cathepsin L is an important enzyme in the endolysosomal entry pathway, and because previous studies, though limited in number, have demonstrated that Z-Tyr-Ala-CHN_2_ interacts with and inhibits cathepsin L and B [50,51], we performed an enzymatic cathepsin L assay to assess whether Z-Tyr-Ala-CHN_2_ acts on cathepsin L. Like the positive control leupeptin and the known cathepsin L blocker K777, Z-Tyr-Ala-CHN_2_ blocked cathepsin L activity with an EC_50_ < 0.05 µM (Figure S3).

**Figure 7:**
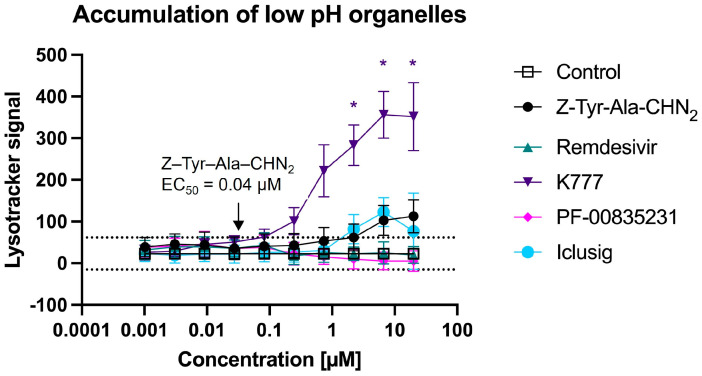
HCI assay with LysoTracker to compare organelle accumulation between different compounds in HeLa-hACE2 cells. The dotted horizontal lines represent the mean ±3 × SD of the LysoTracker signal from the virus control. * is indicative for significant organelle accumulation (adjusted *p* < 0.05) at the corresponding concentration relative to the virus control, as determined by using two-way analysis of variance with Šidák correction for multiple comparisons. The intra-experiment dependency of the data is taken into account. The mean values ± SD are based on four experiments.

It is known that some compounds exert antiviral activity by impairing the endolysosomal pathway in an untargeted manner [52]. As this is undesirable, we wanted to assess whether Z-Tyr-Ala-CHN_2_ impairs lysosomal trafficking. In this manner, we can determine whether its observed in vitro activity results from cathepsin L inhibition or instead results from untargeted disruption of the endolysosomal pathway. To this end, an HCI-based antiviral assay with LysoTracker dye was performed. PF-00835231, remdesivir, and K777 were included in the assay as reference compounds (Figure 7). Since remdesivir and the oral form of PF-00835231 are both approved antivirals for the treatment of COVID-19 infection, and act post entry, we did not expect them to disrupt lysosomal trafficking. Indeed, neither one of the compounds increased the intensity of the LysoTracker signal. K777 significantly increased the intensity of the LysoTracker signal at a concentration of ≥2.2 µM (adjusted *p*-value < 0.05). However, the significant increase only occurred at concentrations that are >1000 times higher than its EC_50_ value of 0.0006 µM (*n* = 4) retrieved from the same assay. Z-Tyr-Ala-CHN_2_ did not significantly increase the intensity of the LysoTracker signal, not even at the highest test concentrations. Hence, the antiviral effect against SARS-CoV-2 of Z-Tyr-Ala-CHN_2_ was not deemed to be resulting from a disruption in lysosome trafficking and instead can be attributed to cathepsin L inhibition. 

## 4. Discussion

Therapeutics are needed to help protect the population and to better prepare for future coronavirus outbreaks. In this study, Z-Tyr-Ala-CHN_2_ was identified as a singleton in a primary phenotypic screen with antiviral activity against SARS-CoV-2 in VeroE6-eGFP cells. Available analogues of Z-Tyr-Ala-CHN_2_ did not have any antiviral activity in VeroE6-eGFP, potentially because they all lack the diazo carbonyl group (RC(O)CH = N^+^ = N^–^) motif that is present in Z-Tyr-Ala-CHN_2_, a moiety that might act as a covalent binder to its target. Further research is warranted to assess if this moiety is indeed responsible for its activity, and whether the presumed covalent binding is due to the carbonyl function or the diazo group (or both). If this proves to be the case, it might be worth performing a diverse chemical space exploration maintaining the diazo carbonyl group, or to explore other covalent binder motifs, to improve compound potency. Indeed, covalent binders have proven valuable within various disease areas, with such binding motifs being present in many marketed drugs, a recent example being the SARS-CoV-2 M^PRO^ inhibitor nirmatrelvir [53].

In viral yield reduction assays, Z-Tyr-Ala-CHN_2_ had sub-micromolar activity (EC_50_ = 0.37 µM) and caused a 4-log drop in virus replication without any signs of toxicity. The observed activity of Z-Tyr-Ala-CHN_2_ was comparable to remdesivir and PF-00835231, two compounds with well-described antiviral activity against SARS-CoV-2 [44,54]. As the COVID-19 pandemic progressed, new variants were detected on a regular basis [55]. Moreover, previous outbreaks with other coronaviruses such as SARS-CoV-1 and Middle East respiratory syndrome coronavirus resulted in more serious illnesses [20]. Therefore, antivirals with broad-spectrum coronavirus activity are desirable. Z-Tyr-Ala-CHN_2_ has activity against SARS-CoV-2, SARS-CoV-1, and HCoV-229E. Moreover, in both HeLa-hACE2 and A549-hACE2 cells, Z-Tyr-Ala-CHN_2_ was more potent than remdesivir, PF-00835231, and molnupiravir against all coronaviruses tested, but was roughly ten times less potent than K777.

Through TOA assays, it was established that Z-Tyr-Ala-CHN_2_ acts in the early steps of the SARS-CoV-2 replication cycle, similarly to the cathepsin L inhibitor K777 and the PIKfyve kinase blocker apilimod [29,56,57]. A pseudotyped virus neutralization assay in VeroE6 cells confirmed an entry mode of action for Z-Tyr-Ala-CHN_2_. Camostat mesylate, a TMPRSS2 blocker, was not active in the pseudotyped virus neutralization assay in VeroE6 cells but was active when the assay was repeated using TMPRSS2-expressing VeroE6 cells. This demonstrates that in VeroE6 cells, entry is not dependent on TMPRSS2, whereas in the TMPRSS2-expressing cells, the TMPRSS2 pathway is preferred. Interestingly, Z-Tyr-Ala-CHN_2_ was not active in TMPRSS2-expressing VeroE6 cells, suggesting that Z-Tyr-Ala-CHN_2_ blocks entry via the endosomal pathway rather than via the TMPRSS2 pathway. Similarly, the cathepsin L inhibitor K777 lost most of its activity when tested on TMPRSS2-expressing VeroE6 cells. This observation is in line with previous studies on SARS-CoV-1 and SARS-CoV-2 [22,29,58,59,60]. One of these studies shows lower activity of K777 in TMPRSS2-expressing Calu-3 and Caco-2 cells than in VeroE6 or HeLa/ACE2 cells [29]. Another study observes lower activity of K777 upon TMPRSS2 expression in A549-hACE2 cells [58]. Yet another study in which 293T-ACE2 cells were infected with SARS-CoV-2 pseudotyped virus demonstrates that the inhibitory activity of the cathepsin L blocker E-64d nearly vanishes upon TMPRSS2 expression [22]. Based on our neutralization assay results, as well as on these previous studies, it becomes clear that overexpression of TMPRSS2 in non-TMPRSS2-expressing cells abolishes dependence on the cathepsin L pathway for SARS-CoV-2 entry and restores sensitivity to TMPRSS2 blockers. Thus, in cell types where TMPRSS2 expression is high, cathepsin L utilization seems minimal, rendering poor efficacy of cathepsin L blockers.

In line with the observed loss of activity in TMPRSS2-expressing VeroE6 cells, Z-Tyr-Ala-CHN_2_ was also inactive against SARS-CoV-2 in Caco-2 cells, a cell type that endogenously expresses TMPRSS2 and that is sensitive to camostat mesylate [22]. Similarly, Z-Tyr-Ala-CHN_2_ did not show antiviral activity in differentiated HNECs infected with SARS-CoV-2, whereas camostat mesylate did. This observation demonstrates that SARS-CoV-2 entry in HNECs is TMPRSS2 dependent, and that endosomal fusion is limited or lacking.

Due to the activity profiles of Z-Tyr-Ala-CHN_2_ and the cathepsin L inhibitor K777 being similar in the TOA assay and in the pseudotyped virus neutralization assay, and because previous reports demonstrated that Z-Tyr-Ala-CHN_2_ targets cathepsin L and B [50,51], an enzymatic cathepsin L assay was performed to assess whether Z-Tyr-Ala-CHN_2_ indeed targets cathepsin L, and thereby exerts its activity within the endosomal pathway. From this assay, it became clear that Z-Tyr-Ala-CHN_2_ is a potent cathepsin L inhibitor with an EC_50_ < 0.05 µM. 

Since Z-Tyr-Ala-CHN_2_ acts on cathepsin L in the endosomal pathway, we assessed whether the compound could also disrupt the endolysosomal pathway in an untargeted manner, as this is a common, but less favorable mechanism of action of many coronavirus inhibitors [52,61,62,63,64,65,66]. Since Z-Tyr-Ala-CHN_2_ did not induce a significant increase in LysoTracker signal (reflective of low pH organelle accumulation), the compound’s in vitro antiviral activity is most likely not a result of endolysosomal pathway disruption and instead can be attributed to cathepsin L inhibition. That said, it is worth considering if Z-Tyr-Ala-CHN_2_ might exhibit dual activity with cathepsin L inhibition being most prominent. This is deemed unlikely since Z-Tyr-Ala-CHN_2_ is relatively potent in cell types where SARS-CoV-2 depends on cathepsin L for entry but remains inactive at high compound concentration in cell types like HNECs and Caco-2 where TMPRSS2 is preferentially used for entry.

Knowing that Z-Tyr-Ala-CHN_2_ is a cathepsin L inhibitor also helps explain why the compound has broad-spectrum activity as, in addition to the S protein of SARS-CoV-1 and SARS-CoV-2, the HCoV-299E S protein can also be activated by TMPRSS2 and cathepsin L. Thus, in cell types where TMPRSS2 expression is minimal or lacking, like HeLa-hACE2, cathepsin L inhibition is expected to prevent infection of HCoV-229E. In line with our results, inhibition of HCoV-229E pseudovirus infection in 293T-CD13 cells was also possible using the cathepsin L blocker K777 [31,67,68].

It is unclear whether targeting cathepsin L can be a valuable therapeutic strategy against SARS-CoV-2 infection since TMPRSS2 expression can abolish dependence on the cathepsin L pathway for virus entry, and because TMPRSS2 is expressed in cell types in the airway that are targeted by SARS-CoV-2 [69,70], namely ciliated cells in the upper respiratory tract and alveolar type 2 cells in the alveoli [71,72]. It is, however, conceivable that the expression of cathepsin L relative to TMPRSS2 throughout the human airway epithelium also has an impact on the entry route. Interestingly, it is reported that cathepsin L protein and mRNA levels are increased in SARS-CoV-2-infected Huh-7 cells, while also, cathepsin L is significantly upregulated in both plasma levels and lungs of COVID-19 patients [73,74]. Moreover, a limited number of in vivo studies exist that demonstrate that cathepsin L inhibition can prevent infection and limit SARS-CoV-2 pathogenesis [26,73]. These findings suggest that cathepsin L could be an important factor during infection and disease progression.

Some research suggests that compared with monotherapy, a combination therapy of a TMPRSS2 blocker and an endosomal pathway blocker could have several advantages, including synergistic antiviral efficacy, fewer side effects due to the lower amounts of drugs used, and potentially, a higher barrier to drug resistance [22,23,27,60,75]. For instance, camostat alone at 10 µM was shown not to fully block infection in TMPRSS2^+^ Calu-3 cells, whereas the combination of 10 µM camostat with 10 µM EST, a broad inhibitor of cysteine proteases, could fully block the infection of SARS-CoV-1 [60]. Moreover, some recent studies suggest that the SARS-CoV-2 Omicron BA.1 and BA.2 variants, which together accounted for most of the new infections in the first half of 2022 [76], preferentially use the endosomal entry pathway instead of TMPRSS2-dependent cell surface entry [77,78,79]. This shift in entry was also described for Omicron infection in HNECs, cells that express both TMPRSS2 and cathepsin L [80]. Surprisingly, Z-Tyr-Ala-CHN_2_ was unable to block entry of the SARS-CoV-2 Omicron BA.1 strain in the HNEC ALI cultures at concentrations up to 20 µM, which suggests that, at least in this model, Omicron entry also makes use of the TMPRSS2 pathway. 

Even though in this work a small molecule screening approach was chosen that resulted in the identification of the cathepsin L inhibitor Z-Tyr-Ala-CHN_2_, when the aim is to combat SARS-CoV-2 through cathepsin L inhibition, antibodies or peptide inhibitors might also hold promise as these can be highly potent and specific [81,82]. Neutralizing antibodies targeting spike have, for instance, already proven to have potential as SARS-CoV-2 therapeutics with many in preclinical and clinical development, [83,84] while several studies have thoroughly investigated peptide inhibitors as SARS-CoV-2 antivirals [85,86].

Studies that further evaluate the in vivo potential of cathepsin L blockers are warranted. One in vivo study in African green monkeys demonstrated that the cathepsin L blocker K777 can limit SARS-CoV-2 pathogenesis and disease severity both in a prophylactic and therapeutic setting [26]. K777 has completed phase 1 clinical trials [29] and recruitment for a phase 2a trial is ongoing to assess its effect on clinical symptoms of COVID-19 infection (ClinicalTrials.gov identifier: NCT04843787). The outcome of this trial will teach us a great deal about the future role of cathepsin L blockers in the treatment of coronavirus infections.

Aside from whether cathepsin L inhibitors have clinical potential against coronaviruses or not, Z-Tyr-Ala-CHN_2_ can serve as a valuable tool compound to study coronavirus entry and replication as it has cell-specific activity, is highly potent, and does not show signs of toxicity.

## Figures and Tables

**Table 1:** EC_50_ and CC_50_ values ± SD and SI values of antiviral agents tested by cellular background and virus type.

		Z-Tyr-Ala-CHN_2_	Remdesivir	PF-00835231	K777	Molnu-Piravir
EC_50_ ± SD (µM)	Assay Type					
VeroE6-eGFP SARS-CoV-2 (*n* = 2)	CPE reduction (GFP)	1.33 ± 0.49	1.42 ± 0.15 (*n* = 4)	>50	0.54 ± 0.14	NT
A549-hACE2 SARS-CoV-2 (*n* = 3)	HCI (spike stain)	0.05 ± 0.003	0.18 ± 0.01	0.18 ± 0.008	0.004 ± 0.0002	NT
HeLa-hACE2 SARS-CoV-2 (*n* = 3)	HCI (dsRNA stain)	0.01 ± 0.001	0.11 ± 0.01	0.16 ± 0.02	0.0006 ± 0.00005	1.01 ± 0.11
A549-hACE2 SARS-CoV-1 (*n* = 3)	HCI (spike stain)	0.05 ± 0.016	0.33 ± 0.02	0.55 ± 0.04	0.003 ± 0.0005	NT
HeLa-hACE2 HCoV-229E (*n* = 2)	HCI (dsRNA stain)	0.07 ± 0.01	0.11 ± 0.009	0.47 ± 0.04	0.002 ± 0.0004	1.32 ± 0.18
Caco-2 SARS-CoV-2 (*n* = 6)	CPE reduction (manual scoring)	>50	0.77 ± 0.26	NT	NT	NT
HNECs SARS-CoV-2 (*n* = 2)	vRNA (qPCR)	>20	NT	0.18 ± 0.14 *	NT	NT
CC_50_ (µM)						
A549-hACE2 (*n* = 2–3)	Toxicity (ATPlite)	>25	>25	>12.5	>0.05	>50
VeroE6-eGFP (*n* = 3)	Toxicity (ATPlite)	>20 (*n* = 3)	>100 (*n* = 3)	>20 (*n* = 2)	~50 (*n* = 3)	>100 (*n* = 3)
SI						
A549-hACE2 SARS-CoV-2		>500	>138	>69	>12.5	NT

*n* is the number of independent experiments on which the EC_50_ or CC_50_ calculations are based. * Oral Pfizer Mpro PF-07321332 was used instead of PF-00835231. CC_50_, half-maximal cytotoxic concentration; CPE, cytopathic effect; dsRNA, double-stranded RNA; HCI, high-content imaging; EC_50_, half-maximal effective concentration; NT, not tested; SD, standard deviation. SI, selectivity index; qPCR, quantitative polymerase chain reaction; vRNA, viral RNA.

## Data Availability

Not applicable.

## Supplementary Materials

The following supporting information can be downloaded at: https://www.mdpi.com/article/10.3390/microorganisms11030717/s1, Figure S1: Structure and activity data (EC_50_ and CC_50_) of Z-Tyr-Ala-CHN_2_ (n = 2) and its analogues (n = 1) derived from the primary screen in VeroE6-eGFP; Figure S2: Z-Tyr-Ala-CH_2_ does not inhibit SARS-CoV-2 infection in an upper respiratory tract model. HNEC ALI cultures were infected with the indicated isolates and treated with PF-07321332 at 2.5 µM (red) and Z-Tyr-Ala-CHN_2_ at 20 µM (black). Cell control (CC; gray bars, DMSO 0.2%); Figure S3: Enzymatic cathepsin L assay demonstrating that both Z-Tyr-Ala-CHN_2_ and K777 inhibit cathepsin L with EC_50_ < 0.05 µM. Leupeptin, an inhibitor of endosomal trypsin-like serine and cysteine proteases, was included as a positive control, whereas water was included as a negative control.
