# Supplementary material for: Identification of Z-Tyr-Ala-CHN2, a Cathepsin L Inhibitor with Broad-Spectrum Cell-Specific Activity against Coronaviruses, including SARS-CoV-2
Source: Microorganisms. 2023 Mar 10;11(3):717. doi: 10.3390/microorganisms11030717 (PMC10055926; doi:10.3390/microorganisms11030717)
Supplement: Supplementary file 1 [file microorganisms-11-00717-s001.zip › microorganisms-2188514-supplementary.pdf]

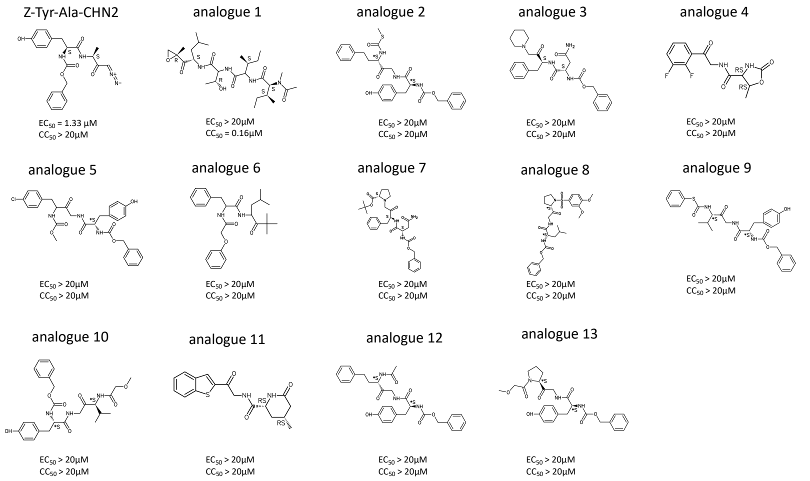

Figure S1. Structure and activity data (EC_{50} and CC_{50}) of Z-Tyr-Ala-CHN_2 (n = 2) and its analogues (n = 1) derived from the primary screen in VeroE6-eGFP cells. EC_{50} values were obtained from a CPE reduction assay against SARS-CoV-2. CC_{50} values were obtained from the toxicity counter screen which was performed on the same cell line using the ATPlite readout. n indicates the number of independent experiments.

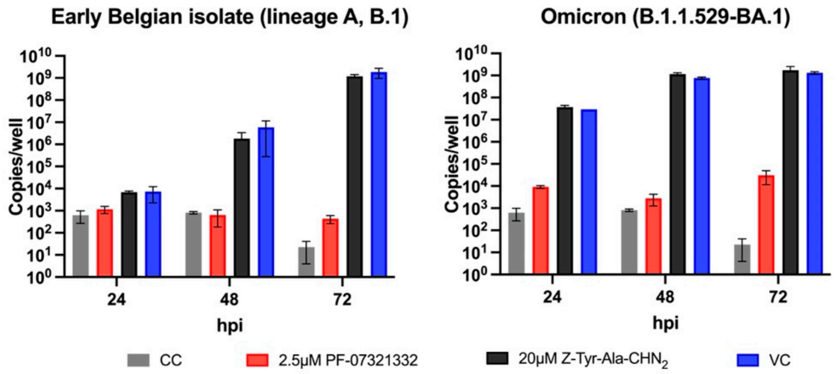

Figure S2. Z-Tyr-Ala-CH_2 does not inhibit SARS-CoV-2 infection in an upper respiratory tract model. HNEC ALI cultures were infected with the indicated isolates and treated with PF-07321332 at 2.5 μM (red) and Z-Tyr-Ala-CHN_2 at 20 μM (black). Cell control (CC; grey bars, DMSO 0.2%). Virus control (VC; blue bars, MOI 0.1 without compound). The mean ± SD from within one representative experiment is shown. Within each experiment, conditions are performed in duplicate. DMSO, dimethyl sulfoxide.

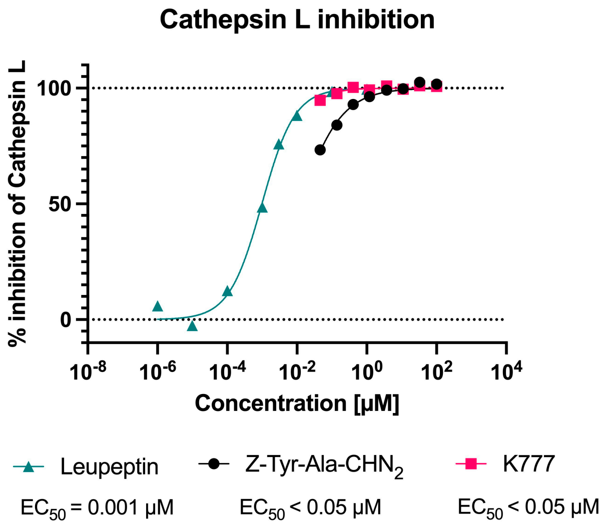

Figure S3. Enzymatic Cathepsin L assay demonstrating that both Z-Tyr-Ala-CHN_2 and K777 inhibit Cathepsin L with EC_{50} < 0.05 µM. Leupeptin, an inhibitor of endosomal trypsin-like serine and cysteine proteases, was included as a positive control, whereas water was included as a negative control. The graph represents one experiment performed to confirm observations from literature.
